# Effect of inulin on the pasting and retrogradation characteristics of three different crystalline starches and their interaction mechanism

**DOI:** 10.3389/fnut.2022.978900

**Published:** 2022-09-08

**Authors:** Xiaolong Ji, Zhiwen Wang, Xueyuan Jin, Zhenpeng Qian, Le Qin, Xudan Guo, Mingsong Yin, Yanqi Liu

**Affiliations:** ^1^College of Food and Bioengineering, Zhengzhou University of Light Industry, Henan Key Laboratory of Cold Chain Food Quality and Safety Control, Henan Collaborative Innovation Center for Food Production and Safety, Zhengzhou, China; ^2^School of Clinical Medicine, Hainan Vocational University of Science and Technology, Haikou, China; ^3^Basic Medical College, Hebei University of Chinese Medicine, Hebei Higher Education Institute Applied Technology Research Center on TCM Formula Preparation, Hebei TCM Formula Preparation Technology Innovation Center, Shijiazhuang, China

**Keywords:** inulin, interaction, physicochemical properties, potato starch, pea starch

## Abstract

At present, there are hardly any studies about the effect of inulin (IN) on the physicochemical properties and structures of different crystalline starches. In this study, three different crystalline starches (wheat, potato, and pea starch) were compounded with natural IN, and its pasting, retrogradation, and structural characteristics were investigated. Then, the potential mechanism of interaction between IN and starch was studied. The results showed that there were some differences in the effects of IN on the three different crystalline starch. Pasting experiments showed that the addition of IN not only increased pasting viscosity but also decreased the values of setback and breakdown. For wheat starch and pea starch, IN reduced their peak viscosity from 2,515 cP, 3,035 cP to 2,131 cP and 2,793 cP, respectively. Retrogradation experiment dates demonstrated that IN delayed gelatinization of all three starches. IN could reduce the enthalpy of gelatinization and retrogradation to varying degrees and inhibit the retrogradation of starch. Among them, it had a better inhibitory effect on potato starch. The addition of IN reduced the retrogradation rate of potato starch from 38.45 to 30.14%. Fourier-transform infrared spectroscopy and interaction force experiments results showed that IN interacted with amylose through hydrogen bonding and observed the presence of electrostatic force in the complexed system. Based on the above, experimental results speculate that the mechanism of interaction between IN and three crystalline starches was the same, and the difference in physicochemical properties was mainly related to the ratio of amylose to amylopectin in different crystalline starches. These findings could enrich the theoretical system of the IN with starch compound system and provide a solid theoretical basis for further applications.

## Introduction

Starch was a macromolecular carbohydrate synthesized by natural plants through photosynthesis, which played an indispensable role in the human diet and industrial applications. Currently, starch is mainly obtained from high-starch-based crops, such as wheat, corn, and potatoes. Natural plant starches are classified into A, B, and C starches according to their X-ray diffraction (XRD) patterns (1). Most cereal starches, such as rice and wheat starch (WS), are A-type starch and show typical characteristic peaks at 2θ = 15°, 17°, 18°, and 23°. Tuber starches, such as potato starch (PoS), are B-type starch, which has characteristic peaks at 2θ = 5.6°, 17.2°, 22.2°, and 24°. C-type starches are a combination of A- and B-type starches with typical characteristic peaks at 2θ = 5.6°, 15°, 17°, and 23° and are commonly found in legume starch and some root starch, such as pea starch (PeS) and taro starch (2). Different types of starches have different crystal structures and granular morphologies, which exhibit different physicochemical properties in their application and processing (3, 4).

However, natural starches have great limitations in food processing due to their thermal instability, pH sensitivity, and retrogradation phenomenon (5). Therefore, the starch modification was extremely necessary in order to better meet the needs of the food industry. In recent years, as a physical modification method, the combination of starch and non-starch polysaccharides has attracted extensive attention at home and abroad due to its convenience, environmental friendliness, and low cost (6). IN, a natural soluble dietary fiber, consisted primarily of D-furan fructose molecules linked by β-(2 → 1) bonds, which has the functions of ameliorating blood sugar, regulating gut microbiota, preventing constipation, and reducing the risk of gastrointestinal diseases (7, 8). IN is widely used in dairy products, bakery products, meat products, and other foods due to its nutritional value, good water-holding capacity, and gelling properties (9).

At present, IN has become a research hotspot in the food field. Luo et al. (10) found that IN with different degrees of polymerization inhibited the long-term retrogradation of wheat starch. Ye et al. (11) observed that IN stabilized the microstructure of the rice gel network and improved the freeze-thaw stability of rice starch gels. In addition, Kou et al. (12) and Krystyjan et al. (13) reached opposite conclusions in the study of the rheological properties of IN on different starch gels. Kou et al. (12) found that moderate amounts of IN reduced the viscoelasticity of wheat gels, while Krystyjan et al. (13) found that IN improved the elastic properties of potato gels. These findings suggested that the type of starch might be the determinant in the rheological properties of IN-starch gels. However, previous studies on the compounded systems of IN and starch investigated the interaction between IN and a single kind of starch, and, until now, there has been no report on the effects of IN on the pasting and retrogradation characteristics and the changes in the crystalline structure of interaction of various types of starches under the same experimental conditions. Starch is classified into A-, B-, and C-type starch according to the crystal pattern, while wheat, potato, and pea starch are the typical representatives of three crystal patterns. Therefore, three types of crystalline starches (WS, PoS, PeS) were selected to be compounded with IN in this experiment. The differences in physicochemical and crystalline structures of the three IN-starch compounding systems were investigated through pasting, retrogradation, XRD, and Fourier-transform-infrared (FT-IR) experiments, and the potential interaction mechanisms between IN and starch were also discussed. The information reported in this study will help further understand the interaction mechanism between IN and starch, which has a good academic frontier. At the same time, the research results can also provide a theoretical basis for the development of starch-based products and the expansion of the market application of IN products.

## Materials and methods

### Materials

The WS, PoS, and PeS were purchased from Henan Enmiao Food Co., Ltd. (Zhengzhou, China). Amylose contents of WS, PoS, and PeS were 31.64%, 24.02%, and 48.82%, respectively, which were obtained according to the method by Doblado-Maldonado, Gomand, Goderwas, and Delcour (14). The IN (content > 88%, degree of polymerization: 2-60, molecular weight, 6,179.36) was purchased from Beneo (Orafti^®^ HSI, Belgium).

Inulin was mixed with starch in the ratio of 1:1 (g/g). Then, 2.5-g IN was dissolved in 25-ml distilled water and stirred for 30 min. Then, the same weight of starch (dry base) was added and stirred again for 30 min to obtain IN-starch suspension.

### Pasting properties

The pasting properties of the IN-starch compound systems were measured by a Rapid Visco-Analyzer (RVA 4500, Perten Instruments, Sweden). The prepared IN-starch suspension was poured into a special aluminum for RVA and measured by the procedure of Liu et al. (1). First, the sample was heated to 50°C and kept for 1 min, and then, the temperature was raised from 50°C to 95°C at a constant rate of 12°C/min and kept at 95 °C for 2.5 min. Later, the temperature was cooled to 50°C at the same rate. Finally, the sample was kept at 50°C for 2 min. The stirring speed was set at 960 rpm in the first 10 s of the test and at 160 rpm for the rest. The pasting parameters, such as pasting temperature (PT), peak viscosity (PV), trough viscosity (TV), final viscosity (FV), setback value (SB), and breakdown (BD) value, were recorded.

### Thermal and retrogradation properties

The differential scanning calorimeter (DSC Q20, TA Instruments Inc., USA) was used to determine the thermal and retrogradation properties of the samples. Approximately 2.5 mg of starch was weighed into an aluminum crucible, and then, 5 μl of IN solution (10%, w/v) was added, sealed, and equilibrated for 12 h at room temperature. The sample was heated by a program that went from 20 to 120°C at a rate of 10°C/min, with a hermetically sealed empty aluminum pan as a reference. The initial temperature (T_0_), peak temperature (T_P_), final temperature (T_C_), and gelatinization enthalpy (ΔH) of the samples were recorded.

The pasted sample trays were stored at 4°C for 7 days. Then, the same procedure was followed to determine the retrogradation parameters. The initial temperature (T_0r_), peak temperature (T_Pr_), final temperature (T_Cr_) and enthalpy of retrogradation (ΔH_r_) of the samples were recorded.

### X-ray diffraction analysis (XRD)

From the RVA experiments, the IN-starch pastes were rapidly dried in a vacuum freeze-drying oven for 72 h by grinding and passing through a 100-mesh sieve to prepare for subsequent experiments.

An appropriate amount of powder was taken to determine the structural characteristics of the IN-starch compound system using XRD (D8 Advance, Bruker Inc., Germany). The testing conditions were a scanning area of 5-35°; a scanning speed of 4°/min; and a sampling step width of 0.02°.

### Fourier-transform-infrared spectroscopy (FT-IR)

The molecular structure of IN-starch compound systems was analyzed by an FT-IR Spectrometer (Vertex70, Bruker Inc., Germany) according to Shi et al. (5). The samples were collected from XRD and mixed with KBr at 1:100 (w/w) ratio. The scanning range was between 4,000 and 400 cm^−1^; the resolution was 4 cm^−1^.

### Interaction force test

The interaction force between IN and starch was determined according to the method of Ren et al. (15) with slight modifications. Approximately 2 g of starch and 1.-g IN (10% starch-5% IN, w/v) were dissolved in 20-ml distilled water. Then, 0, 0.2, 0.6, and 1.0 mol/L sodium chloride or urea was added to the mixture samples. The solution was cooled to ambient temperature after a 20-min water bath at 95°C. Subsequently, the change of the storage modulus (G′) of the blend samples in the frequency range from 1 to 25 Hz was measured using a rheometer (Discovery HR-1, TA Instruments Inc., USA).

### *In vitro* starch digestibility

*In vitro* starch digestion was analyzed according to the method described by Yan et al. (16) with some modifications. Briefly, 0.2 g of the prepared samples from x-ray diffraction analysis were dispersed in a 4-ml sodium acetate buffer (0.1 mol/L, pH 5.2). Then, 1-ml mixed enzyme solution (pancreatin and amyloglucosidase) was added to a 37°C water bath and shaken at 190 rpm to hydrolyze. At 20°C for 120 min, 0.1-ml hydrolyzed fluid was taken and added with 4-ml ethanol (70%) to stop enzymatic digestion. Subsequently, the mixture was centrifuged at 3000 rpm for 10 min. Finally, 0.1 ml of the supernatant was taken, 3-ml GOPOD (D-Glucose Assay Kit) was added at 45°C to a water bath for 20 min, and the absorbance value at 510 nm was tested to calculate the glucose equivalent. Values for rapidly digestible starch (RDS), slowly digestible starch (SDS), and resistant starch (RS) were calculated as follows:


$$\begin{array}{lll} \text{RDS}\left(\text{\%}\right) & = & \left[\left({\text{G}}_{20}-FG\right)/TS\right]\times 0.9\times 100\\ \text{SDS}\left(\text{\%}\right) & = & \left[\left({\text{G}}_{120}-G_{20}\right)/TS\right]\times 0.9\times 100\\ \text{RS}\left(\text{\%}\right) & = & 1-\left(\text{RDS}+\text{SDS}\right) \end{array}$$

where G_20_ and G_120_ were the contents of glucose in the 20 min and 120 min of the enzymatic hydrolysis, respectively. TS was dry basis weight of the samples (g). FG was the amount of glucose hydrolyzed without enzyme addition, FG = 0.

### Statistical analysis

All the experiments were conducted in triplicate. An analysis of variance (ANOVA) was performed by Duncan's test (*p* < 0.05) with IBM SPSS Statistics 26.0 Software Program (SPSS Inc., Chicago, IL, USA). The results were expressed as the mean values ± standard deviations. For all figures, Origin Pro software (Version 9.0, Stat-Ease Inc., Minneapolis, USA) was used.

## Results and discussion

### Pasting properties

The pasting parameters of IN-starch compound system are presented in Table 1, in which it could be clearly observed that there was a significant difference in the influence of IN on the three starches. The addition of IN increased the PT of WS, PoS, and PeS, respectively, from 91.55, 69.18, and 74.30°C to 95.33, 71.95, and 77.98°C, indicating that IN could delay the absorbing and swelling of starch granules during the pasting process. The result may be related to the hygroscopic property of IN, which competed with starch granules for water during pasting, thereby making pasting difficult and increasing PT. Compared to pure starch, PV, TV, and FV values of IN-WS and IN-PeS decreased. The changes in viscosity were reported to be mainly related to the interactions occurring between starch and polysaccharides (17). In this experiment, the decrease in viscosity may be due to the interaction between IN and leached amylose, which formed a protective film and inhibited the swelling of starch granules. The result was consistent with that of a mixed soybean soluble polysaccharide (SSPS)-kudzu and lotus system, which showed that SSPS surrounded the starch granules individually and inhibited the increase in viscosity (18). Unlike the paste viscosity of IN-WS and PeS, the PV and FV of PoS increase after the addition of IN. This phenomenon, probably due to its onset gelatinization, started from the proximal surface of the hila, which was primarily exposing single-helix amylopectin (19). The large amount of amylopectin contributed to the increase in viscosity. The BD value was the difference between the PV and the TV, representing the stability of starch against heat and shear force (8). As seen in Table 1, the DB values of the IN-WS and PeS compound system decreased, which indicated that the addition of IN improved the heat stability of WS and PeS, and this improvement may be attributed to the protection formed by the interaction between IN and amylose. Moreover, the SB value was the difference between the FV and the TV, which indicated the degree of short-term regeneration of starch (20). The SB values showed the same trend as the BD values, which indicated that IN inhibited the short-term recrystallization and rearrangement of amylose of WS and PeS. Similar trends of wheat starch-*Mesona chinensis* polysaccharide mixtures were observed by Liu et al. (21). Compared with the IN-WS and PeS compound system, the BD and SB values of the IN-PoS compound system were increased. The differences in these results may be ascribed to the different crystalline starches that have different crystallinity disruption patterns and the ratios of amylose/amylopectin.

**Table 1:** The pasting parameters of inulin-different crystalline starch compound systems.

**Sample**	**PV (cP)**	**TV (cP)**	**BD (cP)**	**FV (cP)**	**SB (cP)**	**PT (°C)**
WS	2515.00 ± 18.38^b^	1946.50 ± 0.71^c^	568.50 ± 17.68^b^	2919.50 ± 10.61^b^	973.00 ± 11.31^b^	91.55 ± 0.07^e^
WS-IN	2131.00 ± 9.90^a^	1665.00 ± 1.41^b^	466.00 ± 8.49^a^	1937.00 ± 7.07^a^	272.00 ± 5.66^a^	95.33 ± 0.04^f^
PoS	8922.00 ± 89.10^e^	1514.50 ± 113.844^a^	7407.50 ± 24.75^d^	3517.50 ± 3.54^d^	2003.00 ± 117.38^c^	69.18 ± 0.04^a^
PoS-IN	9382.50 ± 47.38^f^	1449.00 ± 11.31^a^	7933.50 ± 58.69^e^	4041.00 ± 28.28^e^	2592.00 ± 39.60^d^	71.95 ± 0.57^b^
PeS	3035.50 ± 26.16^d^	2253.00 ± 26.87^d^	782.50 ± 0.71^c^	4732.00 ± 169.71^c^	2479.00 ± 142.84^d^	74.30 ± 0.42^c^
PeS-IN	2793.50 ± 6.36^c^	2174.50 ± 61.52^d^	619.00 ± 55.15^b^	4043.50 ± 6.36^b^	1869.00 ± 67.88^c^	77.98 ± 0.01^d^

WS, wheat starch; PoS, potato starch; PeS, pea starch; PV, peak viscosity; TV, trough viscosity; FV, final viscosity; BD, breakdown viscosity; SB, setback viscosity; PT, pasting temperature. Values in the same column with different letters were significantly different (p < 0.05).

### Thermal and retrogradation properties

Table 2 shows the gelatinization and retrogradation parameters of the IN-starch compound system. PoS had the highest T_0_ value compared to WS or PeS, which may be due to the difference in the ratio of amylose to amylopectin. That was an important factor in the difference in thermal properties between different starches (22). In addition, PoS has more resistant starch. Several studies reported that there was a positive correlation between resistant starch and thermodynamic parameters (23, 24). The addition of IN to these three starches increased T_0_, T_P_, and T_c_, which was consistent with the RVA test results. ΔH_g_ represented the energy required to disrupt the internal double helix structure of the starch granule, and its value depended on the stability of the crystal structure and the number of crystal regions (25). PoS exhibited the highest ΔH_g_ (15.87 J/g), followed by PeS (10.90 J/g), and then WS (10.07 J/g), reflecting a higher degree of crystallinity of amylopectin in PoS. The addition of IN decreased the ΔH_g_ values of WS, PoS, and PeS. The decrease in ΔH_g_ may be attributed to several reasons. On the one hand, IN competed with starch for the water needed for gelatinization, resulting in incomplete starch gelatinization and ΔH_g_ decrease. On the other hand, IN was a small molecule weight polysaccharide that could easily insert into starch molecules upon heating, leading to weakened stability of part of the crystalline region, thus causing a decrease in ΔH_g_ (26).

**Table 2:** Thermodynamic characteristics of gelatinization and retrogradation of inulin-different crystalline starch compound systems.

**Sample**	**Gelatinization**			
	**T_og_ (°C)**	**T_Pg_ (°C)**	**T_Cg_ (°C)**	**ΔH_g_ (J/g)**
WS	56.95 ± 0.21^a^	63.04 ± 0.47^a^	75.65 ± 0.05^a^	10.07 ± 0.04^b^
WS-IN	59.53 ± 0.09^c^	65.59 ± 0.15^b^	77.86 ± 0.01^b^	8.90 ± 0.10^a^
PoS	61.38 ± 0.03^d^	65.99 ± 0.04^b^	79.91 ± 0.07^c^	15.87 ± 0.09^e^
PoS-IN	64.37 ± 0.15^e^	69.01 ± 0.09^d^	82.32 ± 1.21^d^	15.40 ± 0.30^d^
PeS	58.35 ± 0.11^b^	66.07 ± 0.30^b^	83.40 ± 0.33^d^	10.90 ± 0.06^c^
PeS-IN	61.72 ± 0.20^d^	70.38 ± 0.06^e^	86.06 ± 0.64^e^	9.20 ± 0.00^a^
**Sample**	**Retrogradation**				
	**T**_or_ **(**°**C)**	**T**_Pr_ **(**°**C)**	**T**_Cr_ **(**°**C)**	Δ**H**_r_ **(J/g)**	**R (%)**
WS	42.24 ± 0.04^a^	52.99 ± 003^a^	63.85 ± 0.06^a^	2.48 ± 0.03^a^	24.66 ± 0.11^a^
WS-IN	45.11 ± 0.13^d^	54.98 ± 0.07^b^	64.70 ± 0.21^a^	2.17 ± 0.06^a^	24.39 ± 0.27^a^
PoS	43.48 ± 0.11^b^	60.70 ± 0.09^d^	74.49 ± 0.13^d^	6.10 ± 0.02^e^	38.45 ± 0.13^c^
PoS-IN	44.32 ± 0.49^c^	60.26 ± 1.77^d^	75.35 ± 0.05^d^	4.64 ± 0.39^c^	30.14 ± 1.94^b^
PeS	45.38 ± 0.28^d^	57.36 ± 0.10^c^	70.88 ± 1.07^b^	4.41 ± 0.15^c^	40.51 ± 1.11^c^
PeS-IN	46.09 ± 0.01^e^	58.23 ± 0.14^c^	72.32 ± 0.22^c^	3.64 ± 0.12^b^	39.56 ± 1.36^c^

WS, wheat starch; PoS, potato starch; PeS pea starch; T_0*g*_, the initial temperature of gelatinization; T_*Pg*_, the peak temperature of gelatinization; T_*Cg*_, the final temperature of gelatinization; ΔH_*g*_, the gelatinization enthalpy; T_*or*_, the initial temperature of retrogradation; T_*Pr*_, the peak temperature of retrogradation; T_*Cr*_, the final temperature of retrogradation; ΔH_*r*_, the retrogradation enthalpy; R, retrogradation rate. Values in the same column with different letters were significantly different (p < 0.05).

For recrystallized starches, it was noticed that the T_0r_, T_Pr_, or T_Cr_ for starch paste retrogradation was much lower than those of the native (Table 2). This phenomenon means that the extent of crystalline zones in retrograded samples was looser than that of the native counterparts (27). Similar to the ΔH_g_ results, PoS had the highest ΔH_r_ (6.10 J/g), followed by PeS (4.41 J/g) and WS (2.48 J/g). This difference was related to the ratio of long-chain branches to short-chain branches of amylopectin among different starches (28). The higher the ratio, the higher the crystallinity. The highest ratio was found for B-type starch, the lowest for A-type starch, and C-type starch was in the middle between A and B types (4). The measurements of ΔH_r_ of these three starches were consistent with their polymorphic forms.

The retrogradation rate (R) was the ratio of ΔH_r_ to ΔH_g_, which was positively correlated with the degree of starch retrogradation. The R of the IN-starch compound systems was lower than that of starch alone, which indicated that IN inhibited the regeneration of starch. In this study, the presence of IN reduced the available water in the compound system, which limited the activity of the starch chain (29). Besides, IN as a hydrocolloid could incorporate with the leached amylose and amylopectin molecules through hydrogen bonding, which reduced self-association between either amylose or amylopectin, thereby retarding retrogradation (30).

### XRD analysis

**Figure 1:**
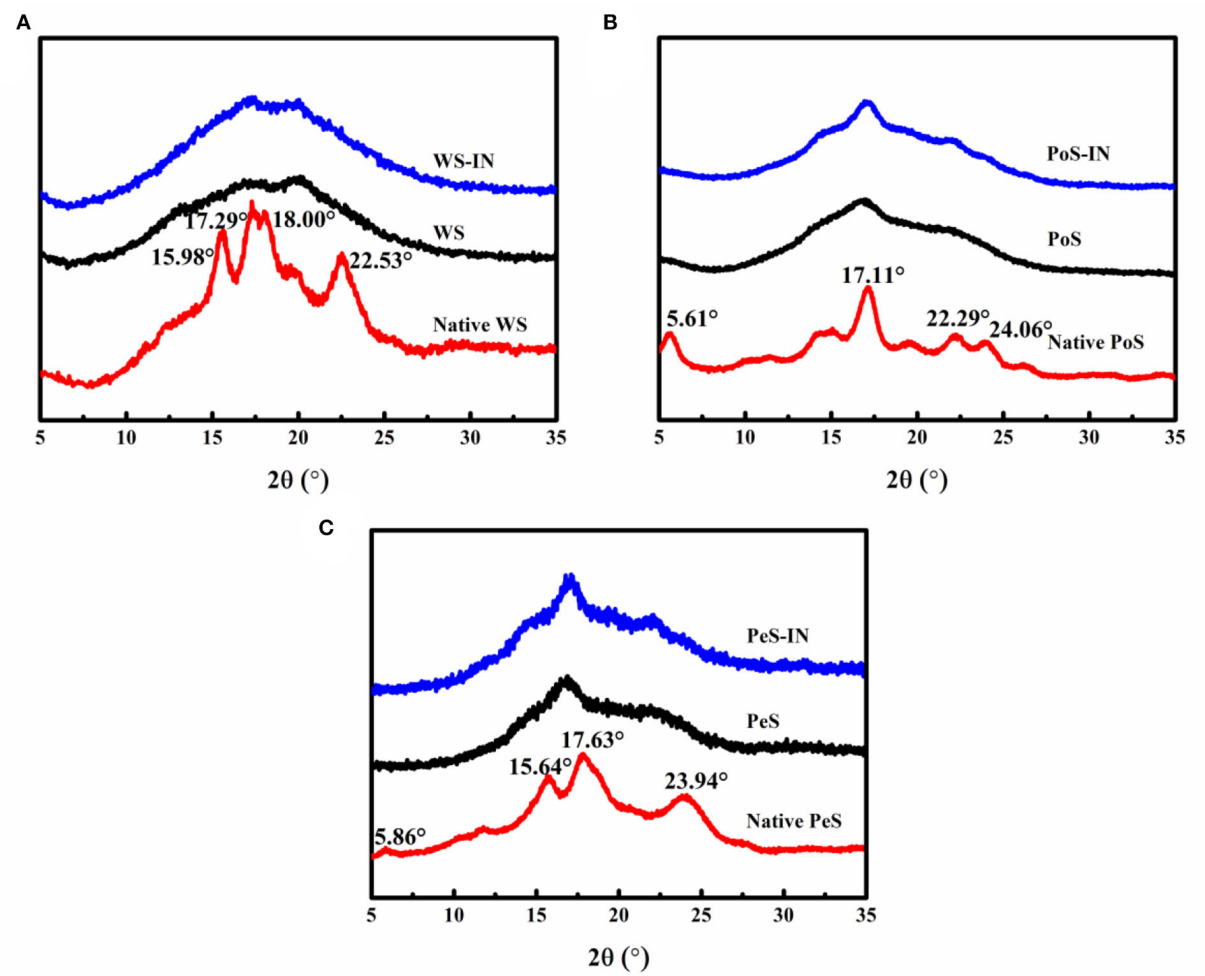
The XRD patterns of the different crystalline starch and inulin-starch compound systems: **(A)** wheat starch, **(B)** potato starch, **(C)** pea starch.

Figure 1 shows the XRD patterns of native WS, PoS, PeS, and IN-starch compound systems. Natural starch displays different diffraction peaks at the same diffraction angles due to the difference in its internal structure. WS had distinct diffraction peaks at 15.58, 17.29, 18.00, and 22.53° with A-type diffraction patterns (Figure 1A); PoS distributed strong diffraction peaks at 5.61, 17.11, 22.29, and 24.06° with B-type diffraction patterns (Figure 1B); PeS showed a C-type diffraction pattern with distinct diffraction peaks at 5.86°, 15.64°, 17.63°, and 23.94° (Figure 1C). The results were in agreement with previous reports (31). After gelatinization, the diffraction peaks of native five starches became blunt and showed a tendency to move to the left, which indicated that gelatinization disrupted the crystal structure and increased the amorphous structure in the starch (32).

The gelatinized WS showed diffraction peaks at 17° and 20°. The IN-WS compound system had not changed the position of the peaks, while it enhanced the diffraction intensity at 17°. The diffraction peaks of the gelatinized PoS appeared at 17° and 22°, and the addition of IN enhanced the diffraction intensity at 17° and 22°. Similar to gelatinized PoS, the same changes were observed at 17° and 22°. The peak shown near 17° for starch was characteristic of amylose (33). The addition of IN made the diffraction peak at 17° more significant for the compound system, which may be due to the interaction of IN with the leached amylose during the pasting process. According to the pasting determination, IN could inhibit the swelling and breakdown of starch as well as improve the stability by heat so that part of the starch granules had better integrity, ultimately enhancing diffraction intensity.

### FT-IR spectroscopy analysis

**Figure 2:**
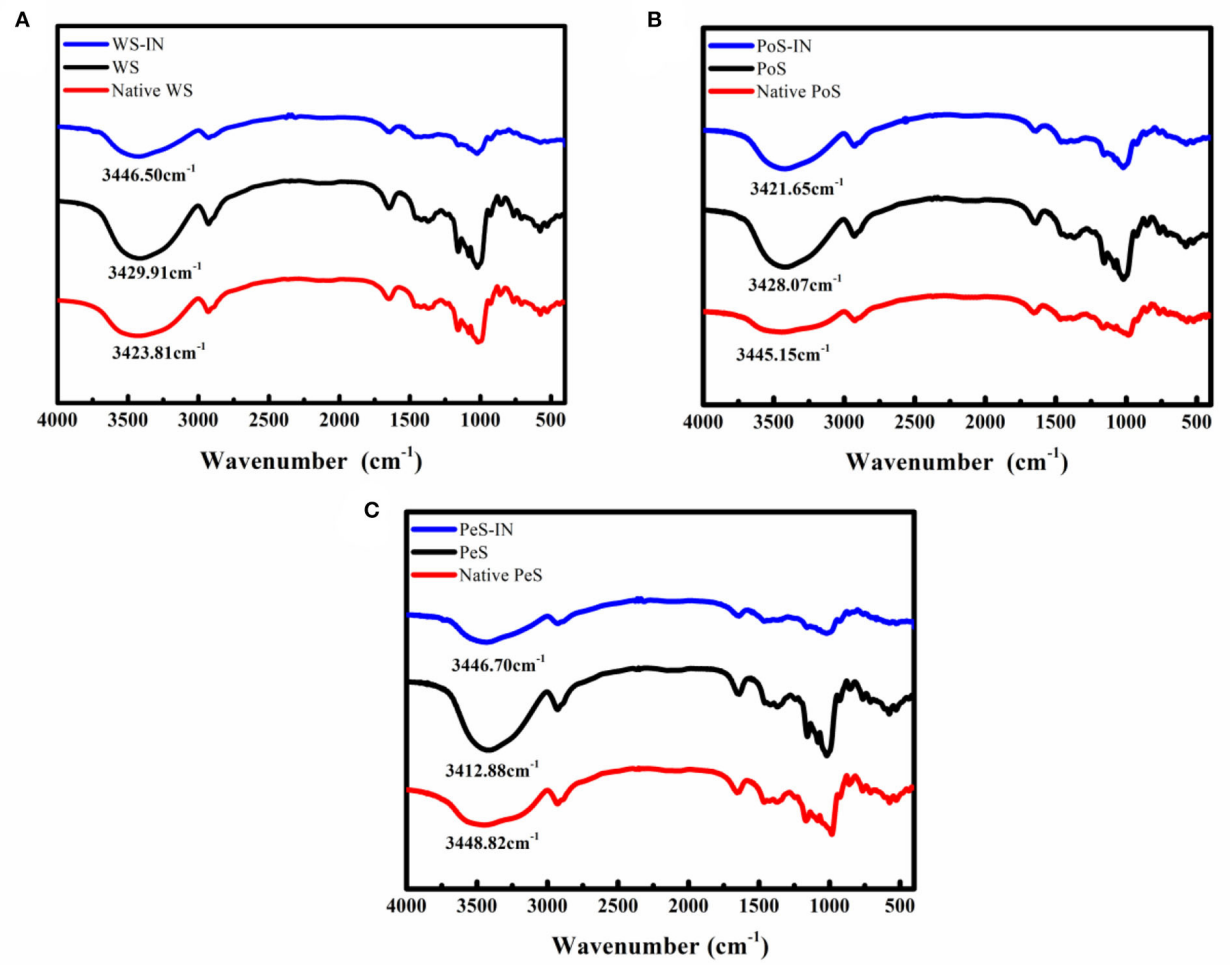
FT-IR spectra of inulin-different crystalline starch compound systems: **(A)** wheat starch, **(B)** potato starch, **(C)** pea starch.

The FT-IR spectra of three types of IN-starch compound systems are presented in Figure 2. Compared with natural starch, no new characteristic peaks appeared in the IN-different crystalline starch compound systems, which indicated that no covalent binding occurred between IN and starch. In addition, the addition of IN only caused changes in the intensity, shape, or position of the absorption peaks, which indicated better compatibility among IN and starch. The positions of characteristic absorption peaks between the different samples are shown in Supplementary Table S1. The band at 3,400 cm^−1^ was ascribed to stretching vibration absorption of associated hydroxyl groups between polymers, which may be intramolecular hydrogen bonds within the same molecule or intermolecular hydrogen bonds between adjacent molecules (34). Notably, the band of the hydroxyl group redshifted with the addition of IN. This result may be explained by the presence of a large number of hydrophilic groups-hydroxyl groups in IN, while starch was a polyhydroxy polymer, and there were also a large number of hydrogen bonds within and between its molecules, eventually resulting in a shift of the absorption peak to higher wave numbers. Meanwhile, 2,900 cm^−1^ was the stretching vibration of C-H, whereas 1,647 cm^−1^ was the bending vibration (35). The band at 1,370 cm^−1^ corresponded to angular vibration of C-H. The absorption peak at 1,160 cm^−1^ and 1,021 cm^−1^ was due to the stretching vibration of C-O (36).

### Interaction force test

**Figure 3:**
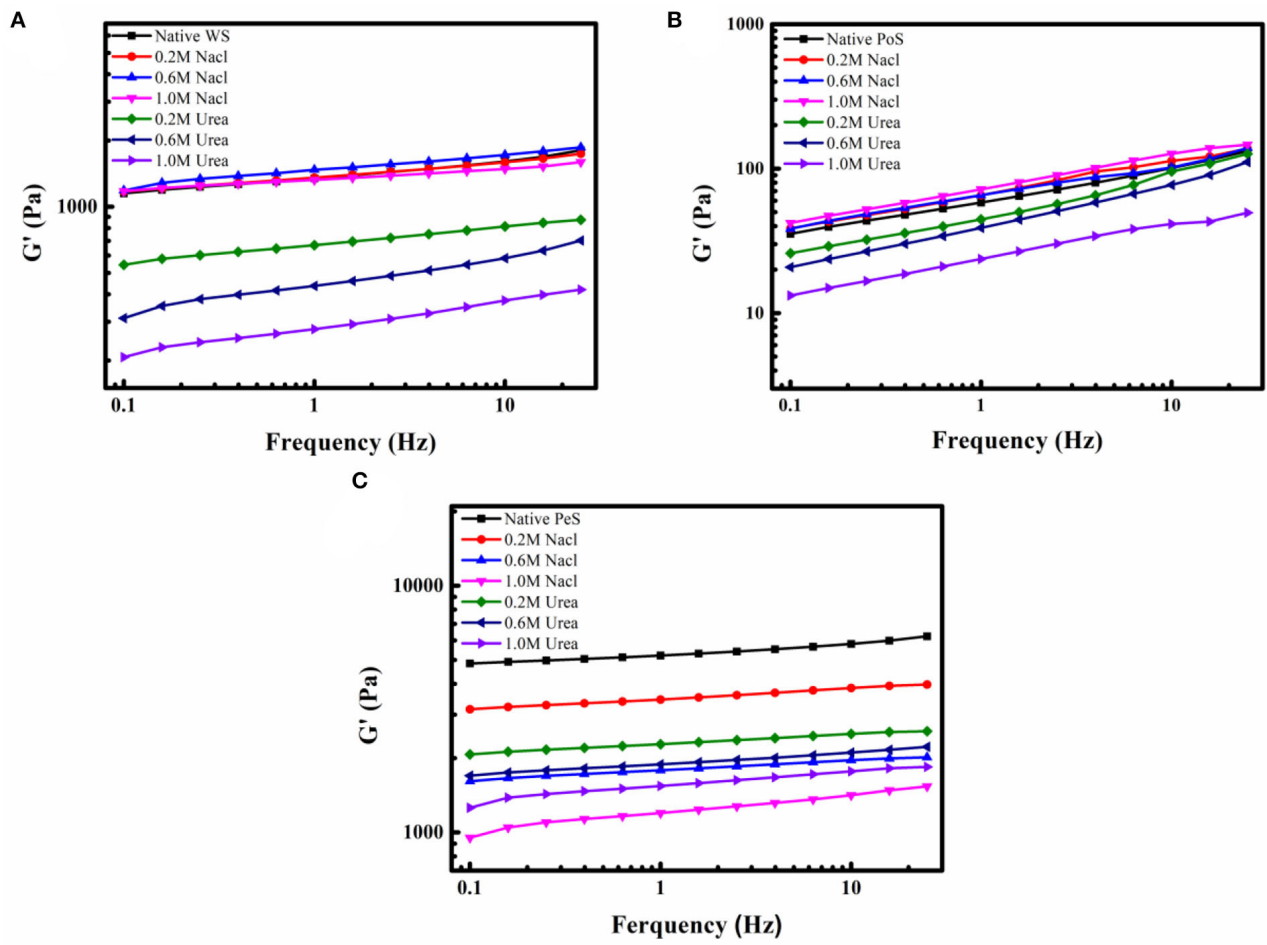
The G′ curves of IN-different crystalline starch compound systems with different concentrations of NaCl and Urea: **(A)** wheat starch, **(B)** potato starch, **(C)** pea starch.

In a previous study, it was analyzed that certain forces exist between IN and starch. From their molecular structure, the interaction forces between non-starch polysaccharides and starches can be observed to include electrostatic forces, hydrogen bonds, hydrophobic interactions, and van der Waals force (37). In order to investigate the forces existing between IN and three different crystalline starches, it was necessary to disrupt the intermolecular forces during the formation of the IN-starch compound systems. Urea and sodium chloride were the commonly used reagents to break intermolecular forces, where urea primarily breaks hydrogen bonding and sodium chloride mainly interferes with electrostatic forces. With the increase in the concentration of urea, the G′ of the IN-WS, PoS, and PeS compound systems kept decreasing, which indicated that urea broke the hydrogen bonding between IN and starch. However, the influence of NaCl on the different compound systems was different. With the increasing concentration of sodium chloride, the G′ of WS and PoS compound systems showed a slightly increasing trend (Figures 3A,B), while PeS compound system kept decreasing (Figure 3C). The increased G′ of the WS and PoS compound system may be accounted for by the formation of an amylose-IN network structure between the IN and the leached amylose and may be wrapped around the surface of the starch granules (38). The decrease in the G′ of the PeS compound system indicated that there was a strong electrostatic force between the IN-PeS system compared to WS and PoS, which was inextricably linked to the presence of a large amount of amylose in PeS. In a word, hydrogen bonding was the main force that maintained the intermolecular interaction between IN and starch, and there was also an electrostatic force between PeS and IN.

### Potential mechanism of interaction between IN and starch

**Figure 4:**
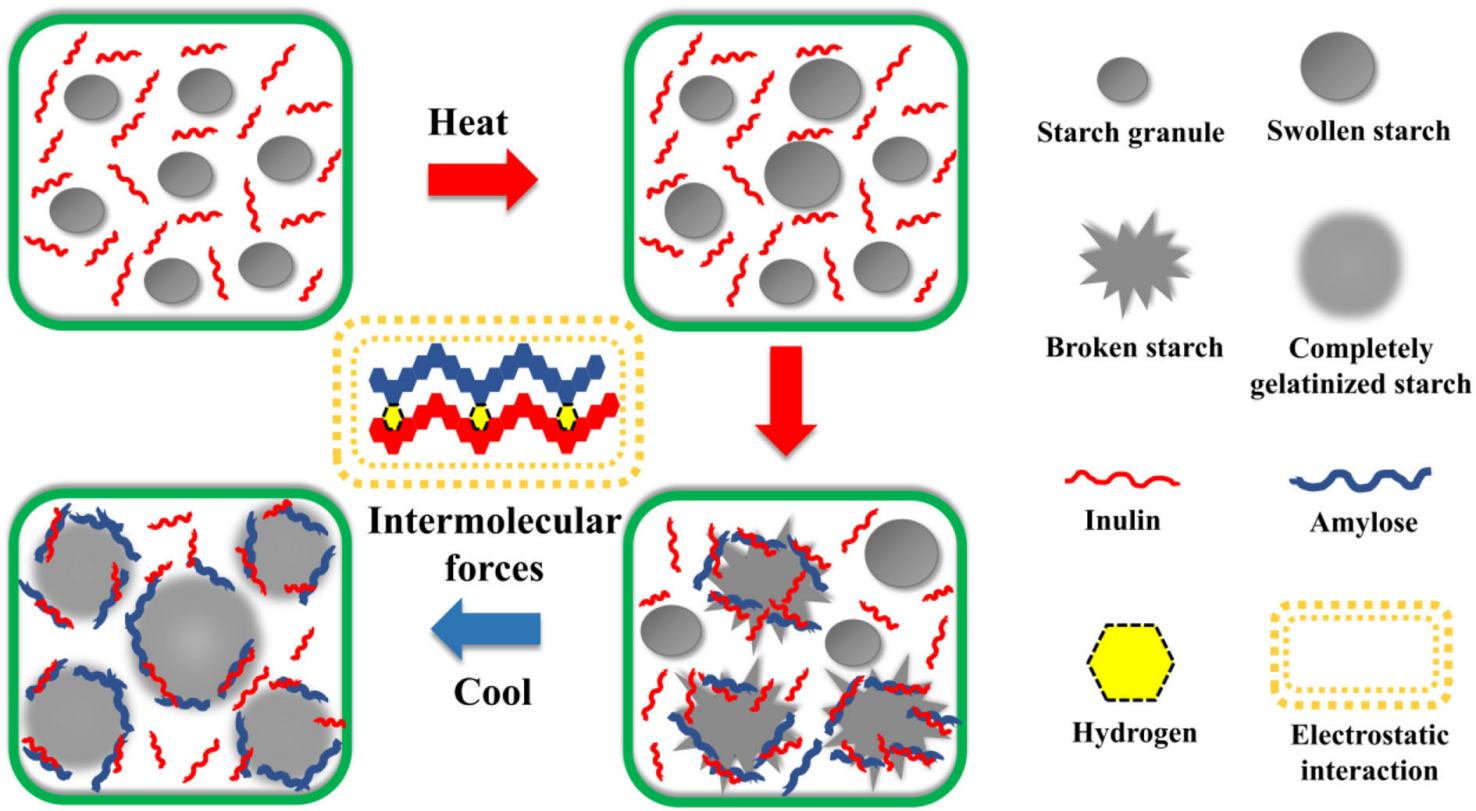
Potential schematics for the mechanism of interaction between starch and inulin.

Based on the above results, a potential mechanism of interaction between IN and starch was speculated (Figure 4). The influence of IN on starch was mainly on the swelling and rearrangement of starch granules during pasting. First, the starch granules were uniformly dispersed in the IN solution. As the temperature increased, the starch granules began to absorb water and swell, resulting in the increase of the volume of starch granules and the increase in the viscosity of the starch paste. At this time, the presence of IN inhibited the swelling of the starch granules and reduced the peak viscosity (RVA test results). When the volume of starch granules swelled to the maximum, the starch granules began to breakdown and the amylose leached out. IN could be combined with the leached amylose by hydrogen bonding and wrapped around the surface of the starch granules, further inhibiting the expansion of the starch granules (as shown by the results of XRD and interaction force experiments). Then, as the temperature decreased, IN could be inserted into the starch molecules and incorporated with the amylose to inhibit the recrystallization of starch. The effects of IN on the three crystalline starch types were both similar and a little different, which mainly depended on different pasting patterns and amylose/amylopectin ratios.

### *In vitro* starch digestibility

Supplementary Table S2 presents the digestion parameters of the different IN-starch compound systems. During the digestion process, IN had the same influence on the change of digestible starch content of the three starches. Among the three starches, the RDS and SDS contents were significantly decreased, while the RS content significantly increased, which demonstrated that IN could inhibit the digestion of starch. This inhibitory effect was the result of a multitude of factors. First, based on the results of RVA experiments, IN could inhibit the swelling of starch granules and protect the integrity of some starch granules, which further inhibited the enzymatic digestion of starch and eventually caused the reduction of RDS content. Second, based on the studies on non-starch polysaccharides-starch systems (39), the polysaccharides could interact with the leached amylose around the starch granules, forming a certain protective effect on the starch, limiting the accessibility of digestive enzymes and enhancing the RD content. The interaction between IN and starch was also observed in XRD experiments so that a similar protective effect may also exist due to IN on starch. Furthermore, the molecular structure of starch, particle size, the amylose/amylopectin ratio, and the interaction between starch and other ingredients may have an influence on the digestibility and degree of starch digestion (40).

## Conclusion

The effects of IN on the physicochemical properties of WS, PoS, and PeS exhibited certain differences. RVA results showed that the presence of IN delayed starch gelatinization and inhibited the swelling and short-term retrogradation of WS and PeS granules. IN significantly decreased the retrogradation rate of the three starches to different degrees, which showed the best inhibition effect on PoS. XRD, FT-IR, and interaction force test results showed that IN mainly interacted with amylose through a hydrogen bond, and there was still an electrostatic interaction between IN and PeS. IN significantly increased the RS proportion in the IN-three starch systems. In addition, the mechanism of action of IN on the three crystalline starch types may be similar. The differences in the physicochemical properties of IN-starch compound systems were related to the starch pasting pattern and the amylose/amylopectin ratio.

## Data availability statement

The original contributions presented in the study are included in the article/Supplementary material, further inquiries can be directed to the corresponding author/s.

## Author contributions

XiJ and ZW contributed to the conception, design, and funding of the study. XuJ, ZQ, and LQ organized the database. XG wrote the first draft of the manuscript. MY and YL contributed to writing, review, and editing. All authors contributed to the article and approved the submitted version.

## Funding

This research was monetarily supported by the Natural Science Foundation of Henan Province (222300420580), the Natural Science Foundation of Henan Province (212300410297), the Science and Technology Basic Research Program of Henan Province (202102110302), and the Doctoral Research Foundation of Zhengzhou University of Light Industry (2020BSJJ015).

## Conflict of interest

The authors declare that the research was conducted in the absence of any commercial or financial relationships that could be construed as a potential conflict of interest.

## Publisher's note

All claims expressed in this article are solely those of the authors and do not necessarily represent those of their affiliated organizations, or those of the publisher, the editors and the reviewers. Any product that may be evaluated in this article, or claim that may be made by its manufacturer, is not guaranteed or endorsed by the publisher.
