# Supplementary material for: Effect of inulin on the pasting and retrogradation characteristics of three different crystalline starches and their interaction mechanism
Source: Front Nutr. 2022 Sep 8;9:978900. doi: 10.3389/fnut.2022.978900 (PMC9493248; doi:10.3389/fnut.2022.978900)
Supplement: Supplementary file 1 [file Data_Sheet_1.docx]

**TABLE S1**. Infrared absorption peaks of Inulin-different crystalline of starch compound systems

| **Sample** | 3400 (cm^-1^) | 2900 (cm^-1^) | 1640 (cm^-1^) | 1370 (cm^-1^) | 1160 (cm^-1^) | 1021 (cm^-1^) |
| --- | --- | --- | --- | --- | --- | --- |
| WS | 3429.91 | 2928.28 | 1647.59 | 1371.81 | 1157.25 | 1021.15 |
| WS-IN | 3446.50 | 2927.55 | 1647.69 | 1374.30 | 1157.48 | 1023.00 |
| PoS | 3428.07 | 2928.65 | 1646.54 | 1369.46 | 1156.83 | 1022.33 |
| PoS-IN | 3421.65 | 2927.83 | 1646.01 | 1370.69 | 1156.44 | 1020.78 |
| PeS | 3412.88 | 2928.72 | 1639.49 | 1370.22 | 1157.07 | 1019.78 |
| PeS-IN | 3446.70 | 2926.43 | 1653.66 | 1362.50 | 1159.18 | 1022.37 |

Values in the same column with different letters were different significantly (*P* < 0.05).

**TABLE S2.** Starch hydrolysis parameters of inulin-different crystalline starch blended systems

| **Sample** | **RDS (%)** | **SDS (%)** | **RS (%)** |
| --- | --- | --- | --- |
| WS | 51.51±0.54^d^ | 20.93±1.94^d^ | 27.56±1.40^a^ |
| WS-IN | 29.25±0.38^b^ | 4.41±0.77^a^ | 66.34±0.39^de^ |
| PoS | 41.05±0.94^c^ | 15.76±0.86^c^ | 43.19±0.08^c^ |
| PoS-IN | 24.05±0.17^a^ | 10.41±0.34^b^ | 65.54±0.51^d^ |
| PeS | 40.82±0.37^c^ | 22.77±0.47^d^ | 36.41±0.84^b^ |
| PeS-IN | 28.37±0.69^b^ | 4.06±0.37^a^ | 67.57±0.32^e^ |

WS, wheat starch, PoS, potato starch, and PeS pea starch. RDS, rapidly digestible starch, SDS, slowly digestible starch, RS, resistant starch. Values in the same column with different letters were different significantly (*P* < 0.05).
